# Innovative approaches to integrating screening, brief intervention, and referral to treatment training into nursing education and practice in Colorado

**DOI:** 10.1186/1940-0640-8-S1-A25

**Published:** 2013-09-04

**Authors:** Leigh Fischer, Elizabeth Pace, Brie Reimann, Carolyn Swenson

**Affiliations:** 1 Peer Assistance Services, Inc., Denver, Colorado, USA; 2 HealthTeamWorks, Lakewood, Colorado, USA

## 

Nurses play a critical role in integrating screening, brief intervention, and referral to treatment (SBIRT) services as standard of care in healthcare settings. Nurses are well positioned to deliver SBIRT services because of their extended patient contact and existing skill sets in health promotion, communication, and patient education. Patients consistently rate nurses in population-wide surveys to be the most trusted professional group in the United States. Data collected from SBIRT initiatives funded by the Substance Abuse and Mental Health Services Administration in the United States indicate the vast majority of SBIRT service providers are nurses, social workers, and counselors. While nurses are key to deliver SBIRT services, specific training and preparation is necessary. This presentation will review evidence and rationale that support nurse-implemented SBIRT and will describe three models employed by the *SBIRT Colorado* initiative to integrate SBIRT into nursing academic preparation as well as nursing practice across diverse settings. The models include: 1) in-person skills-based training; 2) rapid improvement activities; and 3) interactive, online e-learning technology called “SBIRT Substance Use NursingMentor™.” The online training uses computational linguistics, search and streaming media to teach SBIRT skills through engaging simulated interactions. The SBIRT Substance Use NursingMentor was developed as a collaborative effort between MedRespond, Peer Assistance Services, Inc., and NORC at the University of Chicago. Quantitative and qualitative data collected from nurse training participants will be described. Pilot test results from nurses who participated in the online training demonstrated an increase in knowledge, awareness, and understanding of risky drinking, illicit substance use, and related consequences. Data collected from metrics integrated into the online module launched in May 2013 will be analyzed to evaluate the use of e-learning technologies to provide skill-based SBIRT competency training. Presenters will discuss recommendations that indicate how to target and tailor training for nurses for effective SBIRT dissemination.

